# Successful Colonic Stenting Across the Ileocecal Valve With Severe Malignant Stenosis Using Ultra‐thin Scope and Single‐balloon Overtube

**DOI:** 10.1002/deo2.70190

**Published:** 2025-08-25

**Authors:** Takato Maeda, Norihiro Hanabata, Shohei Igarashi, Masayoshi Ko, Koji Shimaya, Hiroshi Numao, Masaki Munakata, Hirotake Sakuraba

**Affiliations:** ^1^ Department of Gastroenterology Aomori Prefectural Central Hospital Aomori Japan; ^2^ Department of Gastroenterology Hematology and Clinical Immunology Hirosaki University Graduate School of Medicine Aomori Japan

**Keywords:** case report, colonic neoplasms, ileocecal obstruction, intestinal obstruction, self‐expandable metallic stent

## Abstract

Self‐expanding metallic stents (SEMSs) are an established palliative option for malignant colonic obstruction, including in cases with proximal lesions. However, SEMS placement across the ileocecal valve (ICV) can be technically challenging because of the anatomical curvature and luminal stenosis. Herein, we report a successful case of colonic stenting for a malignant ileocecal obstruction using an ultra‐thin scope and a single‐balloon overtube. A 72‐year‐old man with alcoholic cirrhosis and multiple liver metastases presented with malignant ileocecal obstruction. Given his inoperability, palliative SEMS placement was attempted. Colonoscopy showed a circumferential tumor in the ileocecum, with no passage of contrast medium into the ileum. Guidewire insertion across the ICV failed because of the inability to visualize the direction of the ileal lumen. To overcome this, we used a rescue technique with an ultra‐thin scope and a single‐balloon overtube. After placing the overtube in the ascending colon, the ultra‐thin scope was advanced through it to explore the stenotic lumen directly. This allowed safe insertion of the guidewire into the proximal lumen of the stenosis. The ultra‐thin scope was withdrawn, and a standard scope was inserted over the guidewire. Finally, a SEMS was deployed across the ICV through the scope. The patient's obstructive symptoms were resolved without complications, and he was discharged 7 days later. This case demonstrates that in cases of malignant ileocecal obstruction where SEMS placement using conventional methods is difficult, a rescue technique using an ultra‐thin scope with a single‐balloon overtube may be a viable alternative.

## 1 Introduction

The placement of self‐expanding metallic stents (SEMSs) for proximal malignant colonic obstruction is regarded as an effective treatment option for bowel decompression [1, 2]. The effectiveness of SEMSs has also been reported in the ileocecal region [3, 4, 5]; however, in cases requiring stent placement across the ileocecal valve (ICV), the stenosis and anatomical curvatures of the structure must be overcome. Here, we report a case of successful colonic stenting for a malignant obstruction in the ileocecal region, which was previously difficult to stent using conventional methods, using an ultra‐thin scope and single‐balloon overtube.

## 2 Case Report

**FIGURE 1:**
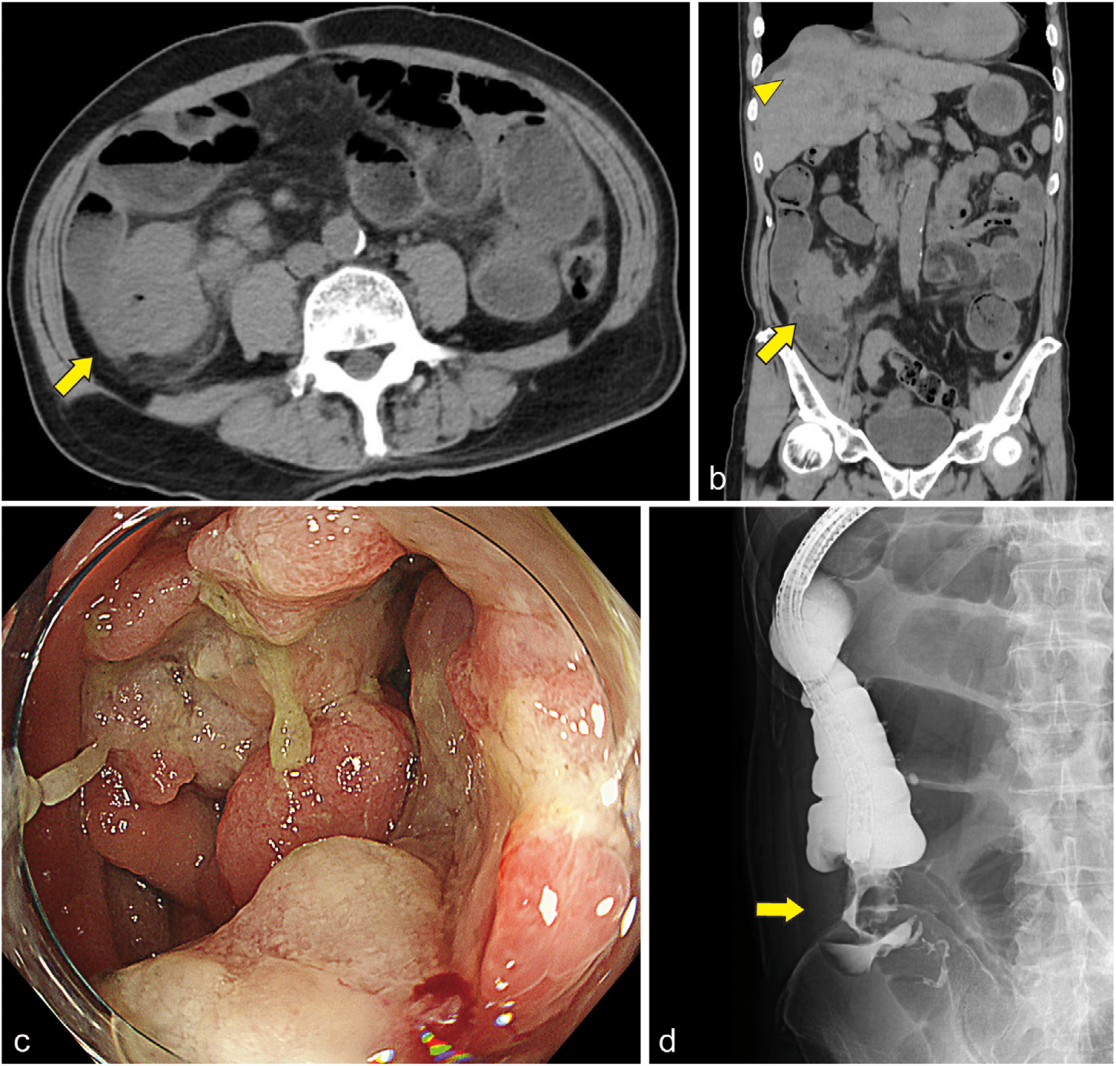
Computed tomography scan showing thickened walls in the ileocecal region with proximal bowel dilatation (arrows). (a) Axial view. (b) Coronal view. The liver shows signs of cirrhosis and metastatic tumors (arrowhead). (c) Endoscopy showing a tumor with severe stenosis in the ileocecal region. (d) Fluoroscopy showing stenosis of the ileocecum, wherein the contrast medium does not flow into the ileum (arrow).

**FIGURE 2:**
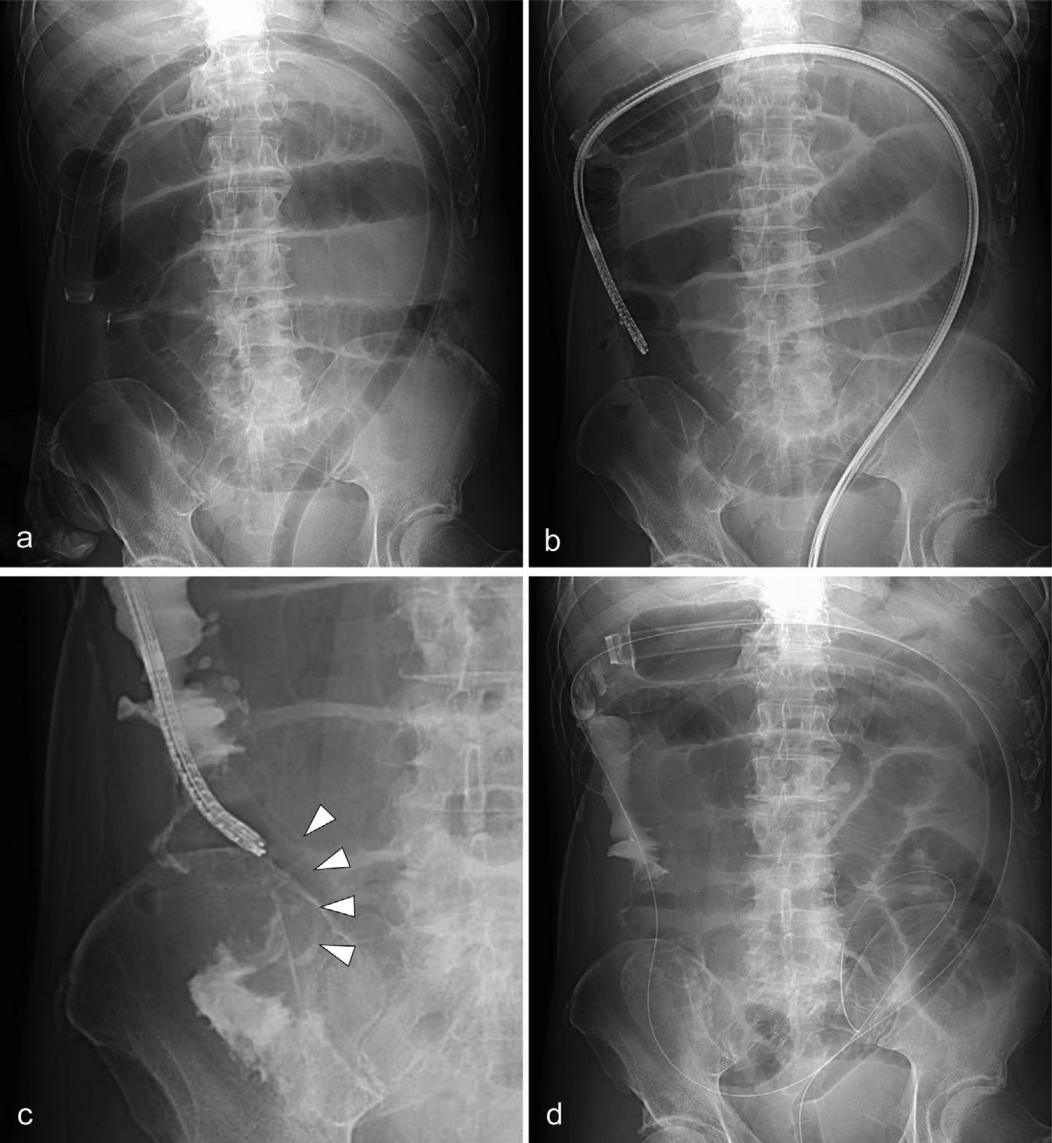
Guidewire delivery to a stenosis using an ultra‐thin scope and single‐balloon overtube. (a) A balloon‐equipped overtube (ST‐CB1; Olympus, Tokyo, Japan) inserted with the PCF‐H290TI (Olympus) is positioned in the ascending colon. (b) An ultra‐thin scope (GIF‐1200N; Olympus) is advanced through the overtube to reach the ileocecal region. (c) The direction to the ileum is identified using the ultra‐thin scope, and a guidewire is advanced to the oral side of the stenosis (arrows). (d) The scope is removed, leaving the guidewire in place.

**FIGURE 3:**
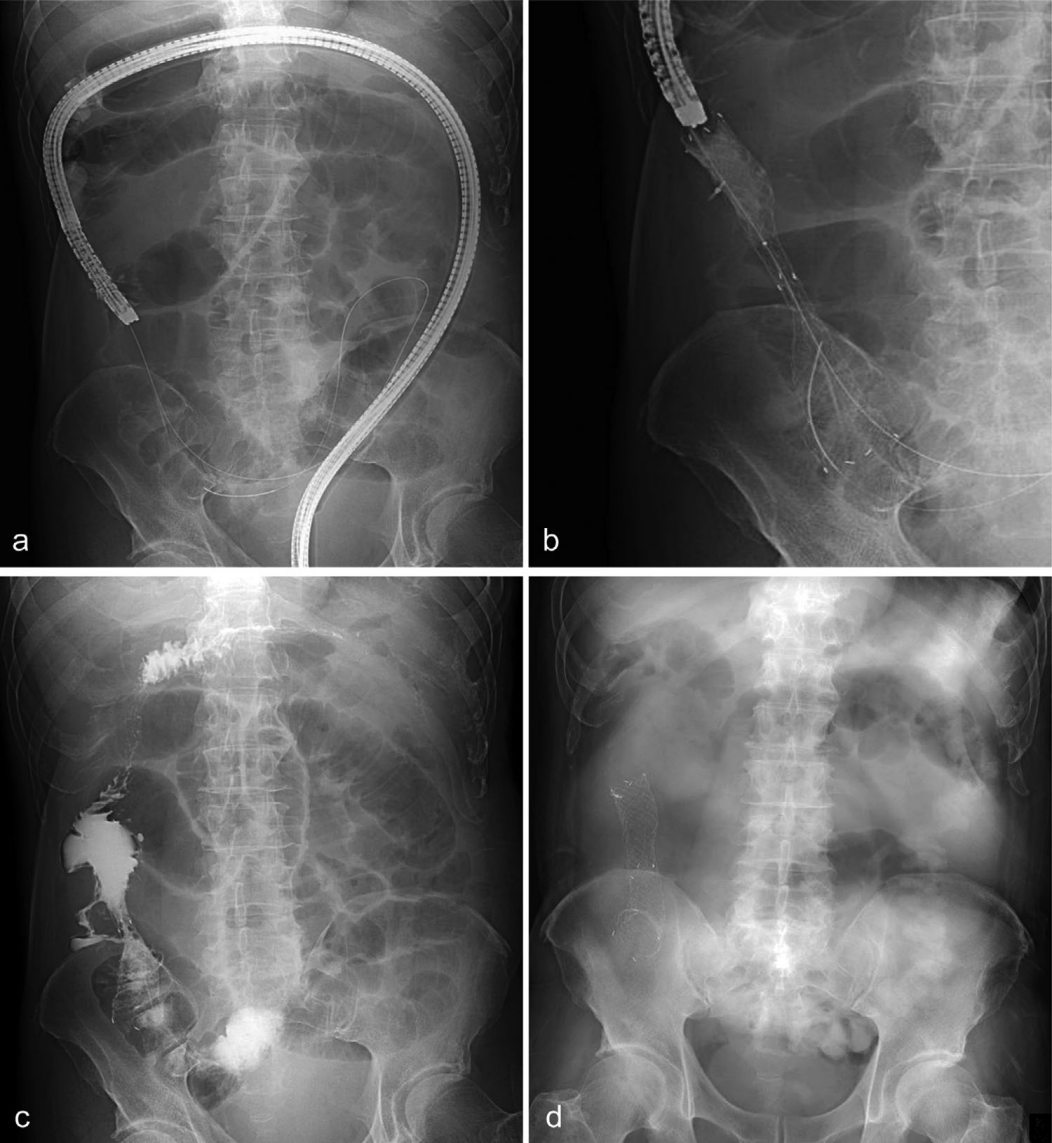
Colonic stenting across the ileocecal valve. (a) The PCF‐H290TI scope (Olympus, Tokyo, Japan) is advanced to the stenosis by following the guidewire. The guidewire is threaded through a pre‐catheter from the channel to the end of the scope. (b) A self‐expanding metallic stent (SEMS) is placed through the scope. (c) Fluoroscopic image after SEMS placement. (d) Abdominal radiograph taken the day after SEMS placement.

A 72‐year‐old man presented to our center with constipation and abdominal distention. Computed tomography (CT) showed a tumor in the ileocecal region and dilatation of the small intestine (Figure 1a,b). Colonoscopy revealed a circumferential tumor with stenosis in the ileocecum (Figure 1c). As per the tumor biopsy results, a diagnosis of well‐differentiated adenocarcinoma was made. CT revealed findings that were suggestive of liver metastasis (Figure 1b), resulting in a final diagnosis of metastatic colorectal cancer with bowel obstruction. The patient's Eastern Cooperative Oncology Group performance status score was 3. Additionally, he had alcoholic cirrhosis (classified as Child‐Pugh B) and cognitive impairment. The patient was deemed ineligible for surgery because of his poor overall health and advanced cancer. Therefore, palliative SEMS placement was attempted. Fluoroscopy showed stenosis from the cecum to the ICV, with the contrast medium not flowing into the ileum (Figure 1d). The SEMS needed to be placed across the ICV; however, the guidewire (Jagwire, 0.035‐inch; Boston Scientific, Marlborough, MA, USA) could not be passed through the ileum via blind manipulation because the direction of the ileum could not be determined. To overcome this challenge, we attempted to evaluate the structure using an ultra‐thin scope. First, a PCF‐H290TI scope (Olympus, Tokyo, Japan) equipped with a single‐balloon overtube (ST‐CB1; Olympus) was reinserted, and the overtube was positioned in the ascending colon (Figure 2a). The scope was withdrawn with the overtube remaining in place, and a second, ultra‐thin scope (GIF‐1200N; Olympus) was inserted through the overtube (Figure 2b). The ultra‐thin scope successfully reached the ileocecum, enabling us to confirm the correct direction of the ileum by observing the stenotic lumen (Figure 2c). The guidewire was successfully inserted into the proximal ileal lumen of the stenosis by manipulating the axis and angle of the scope. Subsequently, the ultra‐thin scope was removed, leaving the guidewire in place (Figure 2d). The PCF‐H290TI scope was then advanced to the stenosis by passing the guidewire through the endoscopic retrograde cholangiopancreatography catheter (Tandem XL; Boston Scientific) that had been inserted from the channel to the tip of the scope (Figure 3a). Finally, a SEMS (HANAROSTENT Naturfit, length: 120 mm, diameter: 22 mm; Boston Scientific) was deployed through the scope across the ICV (Figure 3b,c and S1). The patient's obstructive symptoms improved (Figure 3d), and he was discharged 7 days later. The patient died 2 months later owing to cancer progression; however, there were no complications related to the colonic stent.

## 3 Discussion

Herein, we describe a case of malignant ileocecal obstruction that was difficult to stent using conventional methods but was successfully treated with appropriate SEMS placement using a rescue technique involving an ultra‐thin scope and single‐balloon overtube.

Although there are limited reports on SEMS placement for ileocecal obstruction, it is expected to be as effective and safe as it is for left‐sided colorectal obstruction [4, 5]. However, SEMS placement across the ICV can be challenging owing to technical difficulties caused by its anatomical curvature. Previous studies have reported that guidewires with flexible tips or bendable catheters can effectively pass through angulated stenoses [6]. In the present case, we attempted to use these devices; however, because the proximal lumen of the stenosis could not be confirmed by endoscopy and fluoroscopy, there was a risk of blind insertion of the guidewire, which could lead to perforation. Another treatment option considered was inserting a long intestinal tube to decompress the small intestine, followed by attempting SEMS insertion. However, in this case, the tolerance of a long intestinal tube was poor owing to cognitive impairment. Therefore, we decided to try a rescue technique using an ultra‐thin scope.

The use of an ultra‐thin scope is a viable rescue technique when conventional stenting methods prove difficult [7, 8]. Using an ultra‐thin scope enables precise exploration of the stenotic lumen and facilitates proper insertion of the guidewire. Access to the lesion with an ultra‐thin scope is practical in the rectum or distal colon but difficult in the proximal colon. This is because the ultra‐thin scope is not rigid, which limits the shortening of the colon during scope insertion. To address this issue, we employed a single‐balloon overtube to guide the ultra‐thin scope into the deep colon. Accessing the ileocecum was possible by passing the ultra‐thin scope through the overtube placed in the ascending colon. The guidewire was safely inserted by exploring the stenotic lumen with the ultra‐thin scope, and the SEMS was finally implanted across the ICV through the scope.

The insertion of an ultra‐thin scope into the proximal colon with an overtube has been reported using a double‐balloon endoscopy (DBE) device by Kobayashi et al. [3]. Our approach simplifies the procedure compared to the previous method, potentially allowing SEMS placement to be completed in less time. A reason for this is that, since the overtube of the DBE is longer than the ultra‐thin scope, it must be cut when the ultra‐thin scope is inserted; however, this is not necessary when using a single‐balloon overtube. Additionally, Kobayashi et al. reported that they injected contrast material submucosally into the proximal side of the stenosis to determine the proximal end of the stent. We were able to estimate the length of the stenosis by injecting contrast medium through the ultra‐thin scope. Therefore, this process is not always necessary and can be omitted.

There are several concerns regarding this technique. First, the standard scope must be replaced with a scope equipped with a single‐balloon overtube. This may prolong the procedure and increase the risk of adverse events. In particular, air entering the intestine during the procedure can cause intestinal distension and lead to perforation. Second, few studies have examined SEMS placement across the ICV, and its long‐term efficacy in a palliative setting remains unclear. Further research is needed to determine whether this approach is an effective and safe alternative in challenging cases of SEMS placement in the ileocecal region.

In conclusion, we successfully inserted a SEMS using a single‐balloon overtube and ultra‐thin scope in a case of malignant ileocecal obstruction, wherein conventional methods were difficult. This approach is potentially an effective rescue technique for challenging cases involving severe stenosis or anatomical curvatures of the proximal colon.

## Conflicts of Interest

The authors declare no conflicts of interest.

## Supporting information




**Supporting File 1**: deo270190‐sup‐0001‐Video.mp4.
